# Molecular analysis of small tissue samples obtained via transbronchial lung biopsy using radial probe endobronchial ultrasound

**DOI:** 10.1371/journal.pone.0212672

**Published:** 2019-02-26

**Authors:** Insu Kim, Jung Seop Eom, Ah rong Kim, Chang Hun Lee, Geewon Lee, Eun Jung Jo, Mi-Hyun Kim, Jeong Ha Mok, Kwangha Lee, Ki Uk Kim, Hye-Kyung Park, Min Ki Lee

**Affiliations:** 1 Department of Internal Medicine, Pusan National University School of Medicine, Busan, Korea; 2 Biomedical Research Institute, Pusan National University Hospital, Busan, Korea; 3 Department of Pathology, Pusan National University School of Medicine, Busan, Korea; 4 Department of Radiology, Pusan National University School of Medicine, Busan, Korea; Aix-Marseille Universite, FRANCE

## Abstract

**Background:**

Radial probe endobronchial ultrasound using a guide sheath (EBUS-GS) is used to diagnose peripheral lung cancer. The aim was to identify the accuracy of molecular analysis that were performed with EBUS-GS specimens in patients with non-small cell lung cancer (NSCLC).

**Method:**

From December 2015 to September 2017, we retrospectively studied 91 patients with peripheral NSCLC who underwent surgery after EBUS-GS. Epidermal growth factor receptor (*EGFR*) mutational and anaplastic lymphoma kinase (*ALK*) translocation status obtained from surgical specimens served as the references.

**Results:**

Compared to the reference data, *EGFR* mutational testing of EBUS-GS specimens was in 97% agreement, and the κ coefficient was 0.931 (P< 0.001). In addition, on *ALK* translocation testing, the results of all 91 patients were in agreement with the reference data (concordance rate of 100%, κ coefficient 1.000; P< 0.001).

**Conclusion:**

We found that EBUS-GS could be used for molecular diagnosis, such as *EGFR* mutational and *ALK* translocation status, in patients with peripheral NSCLC.

## Introduction

Lung cancer is the leading cause of cancer-related death worldwide [1]. In recent years, significant developments in the diagnosis and treatment of non-small cell lung cancer (NSCLC) have been made [2,3]. In particular, patient-tailored therapies with epidermal growth factor receptor (*EGFR*) tyrosine kinase inhibitors and anaplastic lymphoma kinase (*ALK*) inhibitors have improved progression-free survival in patients with inoperable NSCLC [4–8].

Patient-tailored therapy requires accurate molecular data, which in turn means that appropriate tissue must be acquired. It is ideal to harvest as much tissue as possible for use in pathologic evaluation and molecular testing. In addition, the remaining tissue should be preserved for further testing [9]. However, due to technical problems with tissue testing, there is a limit to the amount of tissue that can be harvested. [10]. To date, three lung biopsy modalities (surgical wedge resection, percutaneous core needle biopsy [PCNB], and bronchoscopy) have been used for both molecular analysis and histological confirmation [11]. Generally, a prompt and definitive diagnosis using a large amount of tissue can be made on video-assisted thoracoscopic wedge resection under general anesthesia; however, the mortality rate is 0.5% and the complications include persistent air leakage and pneumonia [12]. In addition, although PCNB has afforded good diagnostic performance over many decades, the procedure-related complications include iatrogenic pneumothorax, pleural seeding, and bleeding [13].

Peripheral bronchoscopic techniques, including virtual and electromagnetic navigation, and radial probe endobronchial ultrasound (EBUS) using a guide sheath (GS), have developed rapidly, and are now used to diagnose peripheral lung nodules [14–16]. Recently, transbronchial lung biopsy using a radial probe EBUS and a GS (EBUS-GS) has been shown to afford an acceptable diagnostic yield with a low complication rate [17,18]. However, the accuracy and reliability of molecular analyses of EBUS-GS specimens remain unclear. We retrospectively explored the accuracy of *EGFR* mutational and *ALK* translocation testing in small EBUS-GS tissue samples.

## Materials and methods

### Study population

Between December 2015 and September 2017, we retrospectively accessed the database of the EBUS-GS registry to explore the accuracies of *EGFR* mutational analysis and *ALK* fluorescence in situ hybridization (FISH) status performed on EBUS-GS specimens at Pusan National University Hospital (a university-affiliated, tertiary referral hospital in Busan, South Korea). During the study period, 97 consecutive patients who underwent surgical resection of peripheral NSCLC after a definitive histological EBUS-GS diagnosis were prospectively registered. When evaluating the mutational analyses, the surgical specimens served as the reference samples. Some of our clinical data included previous study conducted but not published [19]. Because of the retrospective nature of the study, the Institutional Review Board of Pusan National University Hospital approved this work without a requirement to obtain informed consent from each subject (approval no. 1711-023-061).

### EBUS-GS procedure

Before each procedure, 4% lidocaine was sprayed into the oropharynx to create local anesthesia and the patient was sedated with intravenous midazolam and fentanyl. First, conventional bronchoscopy using a thin, 4-mm flexible bronchoscope (BF-P260F; Olympus, Tokyo, Japan) was performed to examine the bronchial tree. Next, the bronchoscope was moved as close as possible to the bronchus of interest, guided by the thin-section chest computed tomography (CT) image (0.625mm in both interval and thickness). Then, a radial probe EBUS (UM-S20-17S; Olympus) covered with a GS (K-201; Olympus) was advanced through a 2.0-mm-diameter working channel of the thin bronchoscope to target the peripheral lung lesion precisely. Once the lesion had been accurately identified, the radial probe EBUS was withdrawn, leaving the GS in place to allow brush cytology and forceps biopsy under fluoroscopic guidance [20–23]. Neither virtual bronchoscopy nor electromagnetic navigation was employed [14,15].

### Molecular analyses

Both *EGFR* mutation and *ALK* FISH tests were performed using biopsy tissue and surgically resected samples. *EGFR* mutational tests were performed using an *EGFR* Mutation Detection Kit (PNA clamp; Panagene, Daejeon, South Korea) [24,25]. A commercial *ALK* FISH assay (Vysis *ALK* Break Apart FISH Probe Kit; Abbott Laboratories, Lake Bluff, IL, USA) was used to detect *ALK* translocation [26,27].

### Procedure-related complications

Four hours after EBUS-GS, a plain chest film was taken to detect any procedure-related complication including iatrogenic pneumothorax, and a follow-up chest radiograph was taken the next morning. Severe procedure-related bleeding was defined as a need for intubation, radiological intervention, or transfusion. Any complication such as respiratory failure or pulmonary infection was recorded.

### Statistical analysis

Data are presented as numbers (%) or medians (interquartile ranges [IQRs]) as appropriate. The extents of agreement between *EGFR* mutational tests and *ALK* FISH analyses (EBUS-GS vs. surgical specimens) were determined using Cohen’s κ statistic [28,29]. A two-sided P-value <0.05 was considered to indicate statistical significance. All statistical analyses were conducted using SPSS version 22.0 software for Windows (SPSS Inc., Chicago, IL, USA).

## Results

### Patients

Of the 97 patients who underwent surgical resection of peripheral NSCLC after definitive diagnosis using EBUS-GS, 6 were excluded because their molecular analyses were incomplete. The baseline characteristics of the 91 subjects are shown Table 1. A total of 54 patients were male (59%), and the median age was 67 years (IQR, 60–72 years). The pathological diagnosis was as follows: adenocarcinoma in 68 patients (75%), squamous cell carcinoma in 18 (20%), and NSCLC not otherwise specified in 5 (5%).

**Table 1:** Baseline characteristics of 91 patients who underwent surgical resection after EBUS-GS.

Characteristic	No. (%) or median (interquartile range)
Age, years	67 (60–72)
Male gender	54 (59)
Ever-smoker	45 (50)
Pathological diagnosis	
Adenocarcinoma	68 (75)
Squamous cell carcinoma	18 (20)
Non-small cell lung cancer, NOS	5 (5)

EBUS-GS = endobronchial ultrasound using a guide sheath; NOS = not otherwise specified.

### Molecular analysis

**Fig 1:**
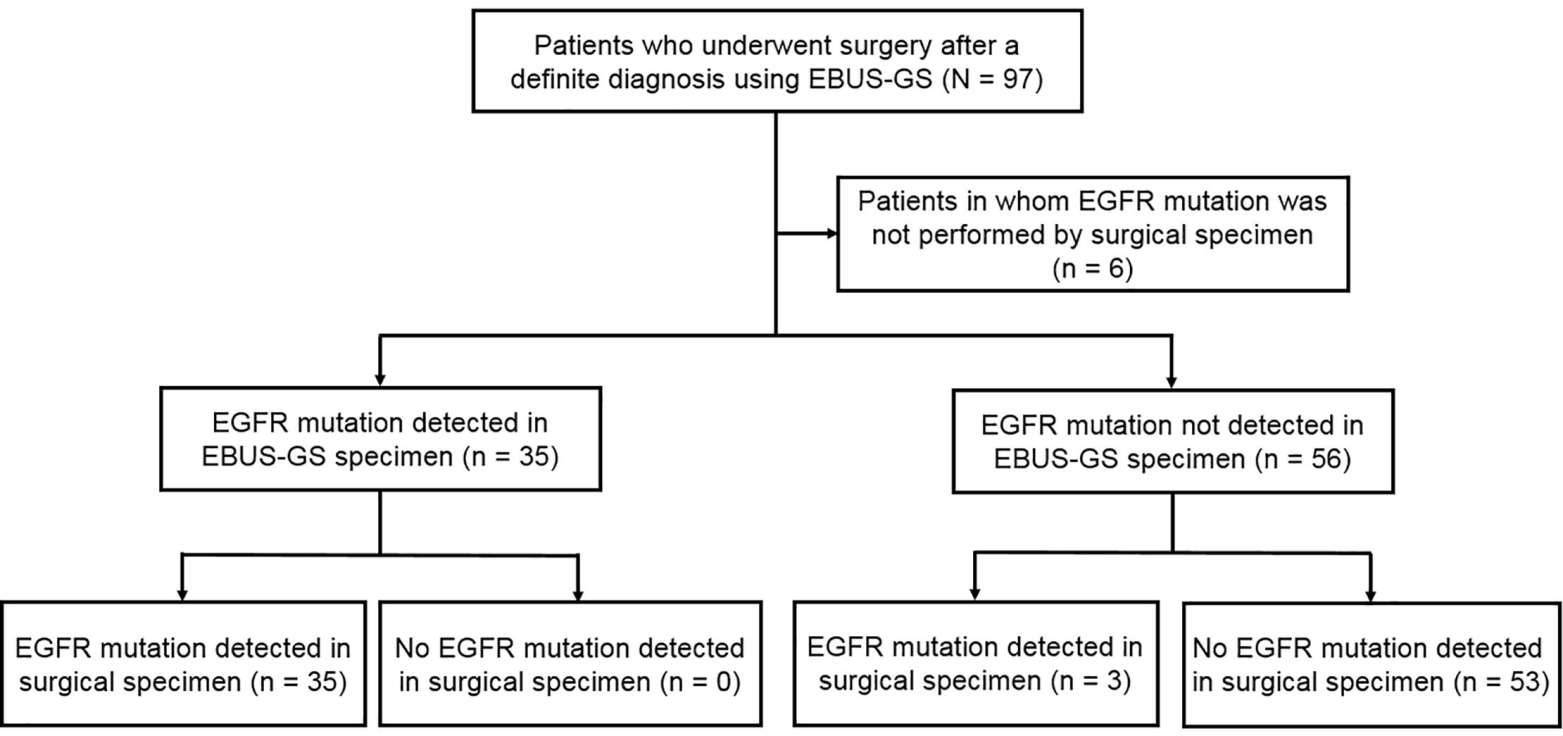
Comparison of *EGFR* mutational analysis between the EBUS-GS and surgical specimens. EBUS-GS = endobronchial ultrasound using a guide sheath; *EGFR* = epidermal growth factor receptor.

**Fig 2:**
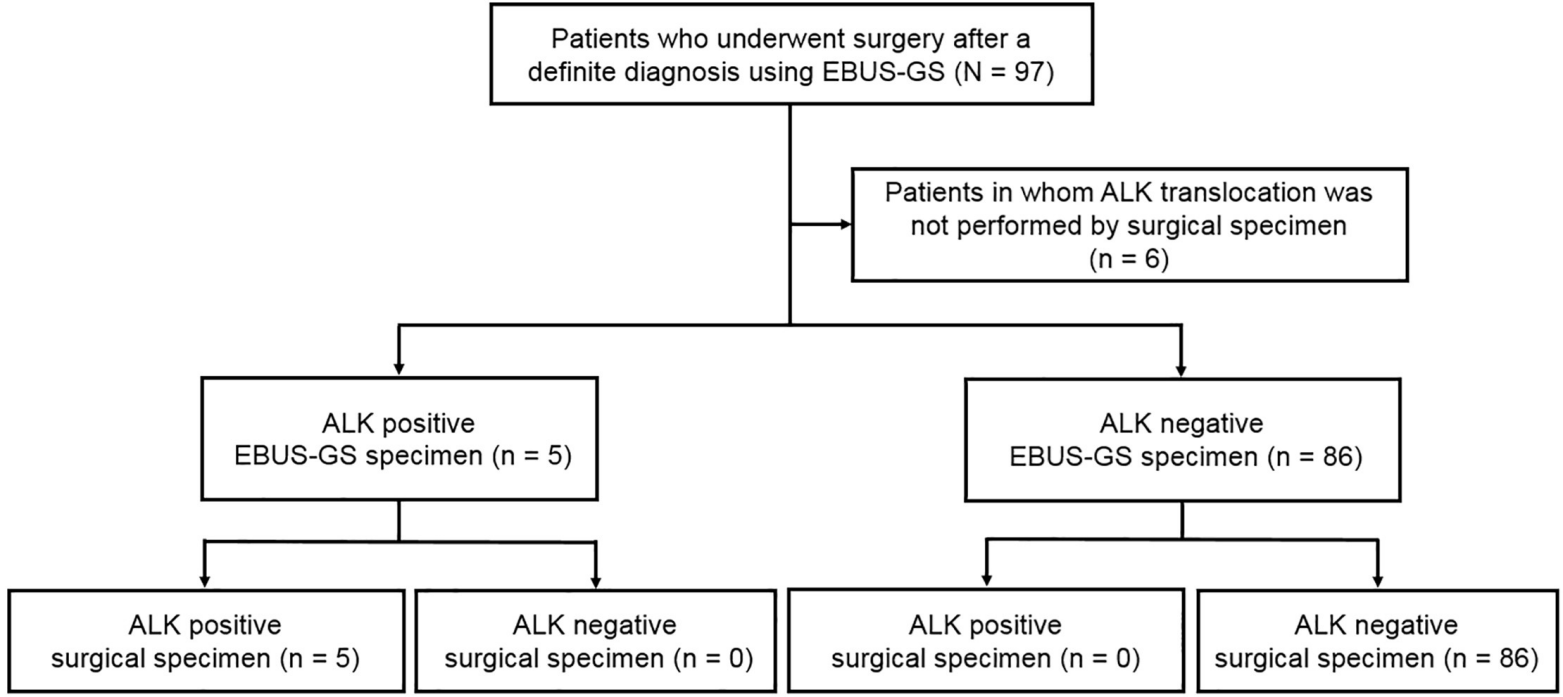
Comparison of *ALK* translocation analysis between the EBUS-GS and surgical specimens. EBUS-GS = endobronchial ultrasound using a guide sheath; *ALK* = anaplastic lymphoma kinase.

Using the EBUS-GS and surgical specimens, *EGFR* mutations were detected in 35 and 38 patients, respectively (38 and 42%). The results differed in three patients (3%) (Fig 1). The agreement rate was 97% and the κ coefficient was 0.931 (P< 0.001) (Table 2). In the *ALK* FISH test, 5 of 91 patients (5%) were positive on both surgical and EBUS-GS testing (Fig 2). The agreement rate was 100% and the κ coefficient was 1.000 (P< 0.001) (Table 2). Additional statistical analysis was performed except for squamous cell carcinoma patients. In 73 patients, the agreement rate of EGFR mutation was 96% and the κ coefficient was 0.918 (*P* = 0.046). In the ALK FISH test, agreement rate was 100% and the κ coefficient was 1.000 (*P*< 0.001).

**Table 2:** Comparisons of the *EGFR* mutational and *ALK* translocation results between the EBUS-GS and surgical specimens.

	Specimens		Correlation analysis		
	EBUS-GS (%)	Surgery (%)	Agreement rate	κ coefficient	*P* value
*EGFR* mutation detected	35/91 (38)	38/91 (42)	97%	0.931	<0.001
*ALK*-positive	5/91 (5)	5/91 (5)	100%	1.000	< 0.001

*EGFR* = epidermal growth factor receptor; *ALK* = anaplastic lymphoma kinase; EBUS-GS = endobronchial ultrasound using a guide sheath.

### Procedure-related complications

Only one patient (1%) developed procedure-related pneumothorax, but recovered spontaneously without chest tube insertion. No other complications were observedno severe hemorrhage, pulmonary infection, or respiratory failure was noted.

### Disagreements in *EGFR* mutational analysis

**Fig 3:**
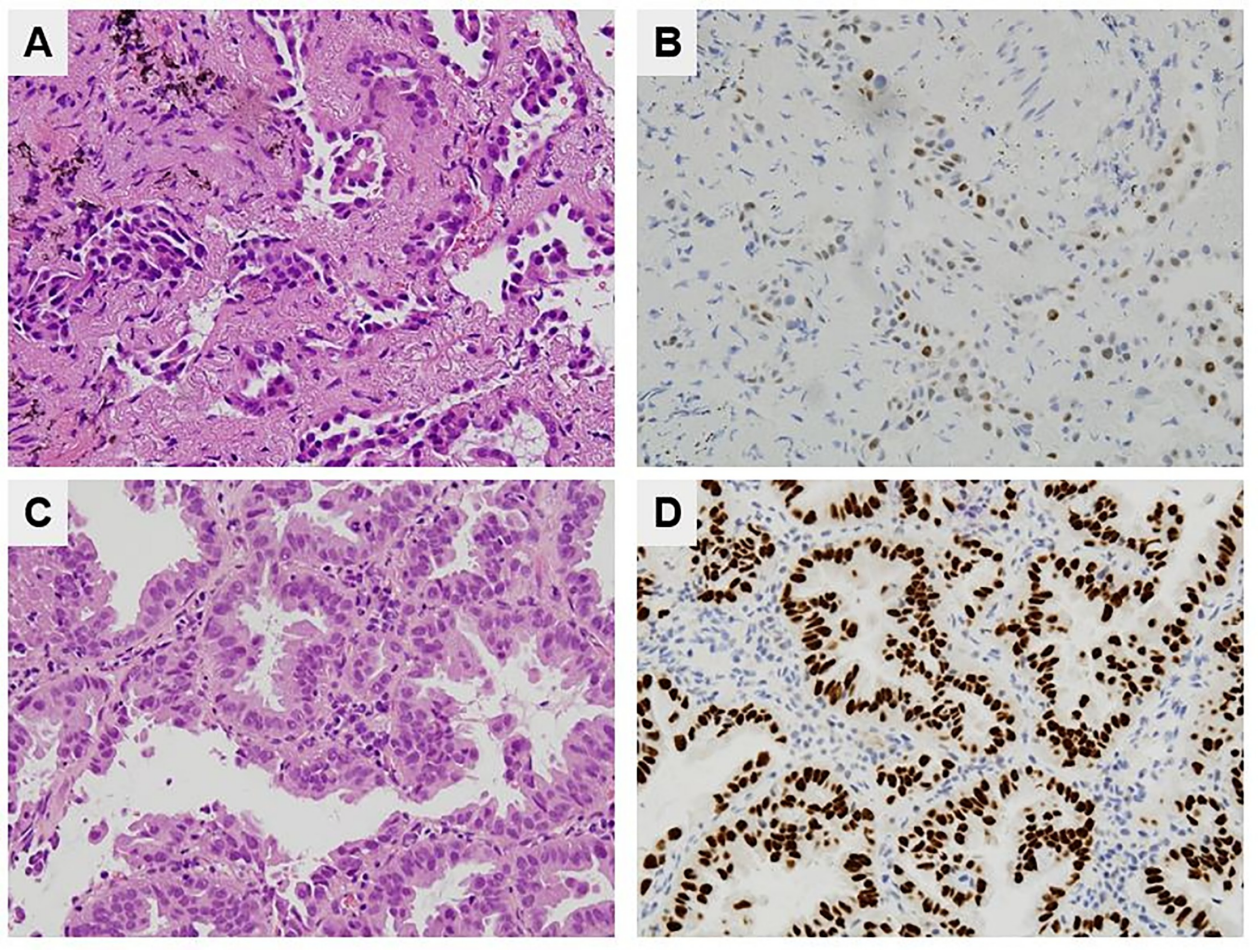
Comparison of an EBUS-GS specimen yielding false-negative *EGFR* results and a specimen yielding correct *EGFR* results. (A) A few adenocarcinoma cells were clustered in the EBUS-GS specimen with the false-negative *EGFR* result (H&E stain, ×400). (B) The EBUS-GS specimen with false-negative *EGFR* result was weakly immunoactive for TTF-1 (×400). (C) Larger numbers of tumor cells were evident in the specimen yielding correct *EGFR* results (H&E stain, ×400). (D) The EBUS-GS specimen with correct *EGFR* result was strongly immunoactive for TTF-1(×400). EBUS-GS = endobronchial ultrasound using a guide sheath; *EGFR* = epidermal growth factor receptor; TTF-1 = thyroid transcription factor-1.

All three patients with inconsistent *EGFR* mutational results were pathologically diagnosed with adenocarcinomas. Compared to the EBUS-GS specimens that yielded correct results, the tumor cell numbers estimated by pathologists were lower on hematoxylin-and-eosin-stained slides of all incorrectly diagnosed EBUS-GS specimens. Moreover, only a few thyroid transcription factor-1-stained cells were observed in two of these EBUS-GS specimens (Fig 3A and 3B); one specimen could not be stained because the available tissue was insufficient (Case No. 2, Table 3). Higher numbers of thyroid transcription factor-1-and hematoxylin-and-eosin-stained cells were observed, at the same magnification, in EBUS-GS specimens that were correctly diagnosed (Fig 3C and 3D).

**Table 3:** Cases with discordant *EGFR* mutational results between the EBUS-GS and surgical specimens.

Case No.	Age, years	Sex	Pathology	Lesion size, mm^a^	Location	Bronchus sign	Probe location	TTF-1 IHC
1	78	Male	ADC	36	RLL	Positive	Adjacent to tumor	Positive
2	74	Female	ADC	48	RUL	Positive	Within tumor	Insufficient^b^
3	70	Female	ADC	38	RML	Positive	Within tumor	Positive

*EGFR*, epidermal growth factor receptor; EBUS-GS, endobronchial ultrasound using a guide sheath; TTF-1, thyroid transcription factor-1; IHC, immunohistochemistry; ADC, adenocarcinoma; RLL, right lower lobe; RUL, right upper lobe; RML, right middle lobe.

^a^ Largest tumor diameter.

^b^ Insufficient EBUS-GS material for TTF-1 staining

## Discussion

We found that EBUS-GS afforded very accurate *EGFR* mutational and *ALK* FISH diagnoses in NSCLC patients. To the best of our knowledge, this is the first report on the accuracy of molecular diagnosis using such specimens. In the 91 NSCLC patients, the accuracies of the *EGFR* mutational and *ALK* translocation tests were 97% and 100%, respectively. Our findings imply that appropriate decision-making in terms of anti-cancer drug selection (*EGFR* tyrosine kinase inhibitors, *ALK* inhibitors, or intravenous cytotoxic chemotherapy) is possible based on molecular data obtained from EBUS-GS specimens of patients with advanced NSCLC.

Tam *et al*. found that 83% of PCNB samples were suitable for molecular testing in 151 patients with NSCLC [30]. However, procedure-related complications occurred in 16% of the patients, of whom 57% required chest tube insertion to manage iatrogenic pneumothorax. Vanderlaan *et al*. reported that the accuracy of molecular analysis using PCNB samples was lower than noted in a previous study [31]; the accuracies of *EGFR* mutational and *ALK* FISH tests performed on PCNB samples were 68% and 65%, respectively, in 22 patients with NSCLC. Chen *et al*. found that all PCNB samples examined could be used for *EGFR* mutational testing [32]. However, this study feature relatively small group of 17 patients, and complications such as pneumothorax (18%) and hemoptysis (12%) were relatively common. In summary, although molecular diagnosis using PCNB samples is reliable, the incidence of procedure-related complications, such as iatrogenic pneumothorax, is relatively high.

In contrast, Steinfort *et al*. showed that EBUS-GS afforded similar pathological diagnostic accuracy compared with PCNB (87.5% vs. 93.3%, respectively) and good sensitivity (86% vs.92%, respectively), associated with considerably fewer procedure-related complications (3%vs. 27%, respectively) [33]. Hamaya *et al*. reported that the overall complication rate of EBUS-GS (pneumothorax or pneumonia) was 1.3% in 965 study subjects [17]. In the present study, the accuracies of *EGFR* mutational and *ALK* FISH testing were 97% and 100%, and the overall complication rate was only 1%. Thus, EBUS-GS is safe and reliable, and the tissue samples can be used for both pathological and molecular analyses.

Generally, molecular analysis proceeds using the tissue that remains after histological examination featuring hematoxylin-and-eosin staining. Therefore, molecular tests are usually performed employing less tissue than in histological examinations and molecular analysis of a small biopsy sample, such as that of PCNB or EBUS-GS, could yield false-negative results because of insufficient tumor tissue or a low tumor fraction. Eberhard *et al*. suggested that the tumor sectional area should be ≥1–2mm, except in non-tumor areas [34]. In addition, in terms of cell counts, >100 tumor cell nuclei should be assessed in terms of FISH. Lindeman *et al*. recommended that mutated cells should constitute ≥20% of all cells when *EGFR* mutational and *ALK* translocation statuses are evaluated [35]. In the present study, false-negative *EGFR* mutational data were obtained from three EBUS-GS specimens (3%). The tumor cell numbers in these specimens were lower than those of other specimens. Thus, insufficient tumor tissue available after histological examination explained the false-negative results. If the *EGFR* mutational status of an EBUS-GS specimen is negative, a false-negative should be considered when the sample volume is small, particularly if the patient is at risk of *EGFR* or *ALK* mutation (has an adenocarcinoma, is a female East Asian, or is a never-smoker) [36–39].

Our study had several limitations. First, although we used an EBUS-GS registry, selection bias may have occurred. Second, this was a single-center study with a relatively small number of subjects; our results can thus not be generalized to other institutions or geographical areas. Third, as we used data from surgical specimens as references, only patients with early-stage lung cancer who underwent surgery were included. Molecular analyses, such as *EGFR* mutational and *ALK* FISH tests, are required by patients with advanced NSCLC to guide the selection of anti-cancer drugs (*EGFR* tyrosine kinase or *ALK* inhibitors). Generally, patients with advanced NSCLC requiring molecular analysis have larger and more tumors than patients with early-stage lung cancer. Previous studies found that the accuracy of EBUS-GS evaluation was associated with lesion size [15,16,40]. Therefore, the accuracy of molecular diagnosis would be expected to be higher in actual clinical practice. Fourth, NGS data were unavailable in the present study. However, the sensitivity and specificity of PNA clamping are 97% and 100%, respectively, similar to the respective values of 95.83% and 98.11% for NGS [41–43]. If NGS is available for both small biopsy samples and surgical specimens, it is possible to compare the concordance of various kinds of mutational analyses. Fifth, given the retrospective nature of this study, it was not possible to quantitatively analyze the effect of sample volume on the molecular data. Generally, the cellularity of the specimen is important for interpretation of mutation analysis results [44]. Recent guidelines recommend mutation analysis of samples with an at-least 20% malignant cell content [35]. To address these issues, an additional prospective multicenter study with a large number of patients that incorporates methods to evaluate cellularity is needed.

## Conclusion

The results of EGFR mutation and ALK gene rearrangement tests on EBUS-GS samples showed good agreement with those on surgical specimens of NSCLC patients.

## Supporting information

S1 Table Clinical data of total patients. ADC, adenocarcinoma; SqCC, squamous cell carcinoma; NSCLC, non-small cell lung cancer; ALK, anaplastic lymphoma kinase; EGFR, epidermal growth factor receptor; RLL, right lower lobe; RUL, right upper lobe; LUL, left upper lobe; LLL, left lower lobe; RML, right middle lobe.
(XLSX)
